# p27^Kip1^ – p(RhoB)lematic in lung cancer‡

**DOI:** 10.1002/path.5218

**Published:** 2019-02-04

**Authors:** Silvio R Podmirseg, Jonathan Vosper, Ludger Hengst

**Affiliations:** ^1^ Division of Medical Biochemistry, Biocenter Innsbruck Medical University Innsbruck Austria

**Keywords:** lung cancer, NSCLC, p27^Kip1^, CDKN1B, RhoB

## Abstract

Lung cancer is the leading cause of cancer mortality worldwide, with adenocarcinomas of the non‐small cell lung carcinoma (NSCLC) subtype accounting for the majority of cases. Therefore, an urgent need exists for a more detailed dissection of the molecular events driving NSCLC development and the identification of clinically relevant biomarkers. Even though originally identified as a tumour suppressor, recent studies associate the cytoplasmically (mis)localised CDK inhibitor p27^Kip1^ (p27) with unfavourable responses to chemotherapy and poor outcomes in NSCLC, supporting the hypothesis that the protein can execute oncogenic activities. In a recent issue of *The Journal of Pathology*, Calvayrac and coworkers uncover a novel molecular mechanism that can explain this oncogenic role of p27. They demonstrate that cytoplasmic p27 binds and inhibits the small GTPase RhoB and thereby relieves a selection pressure for RhoB loss that is frequently observed in NSCLC. This is supported not only by studies with genetically modified mice, but also through identification of a cohort of human lung cancer patients with cytoplasmic p27 and continued RhoB expression, where this signature correlates with decreased survival. This not only establishes a potentially useful biomarker, but also provides yet another facet of the complex roles p27 undertakes in tumourigenesis. © 2018 The Authors. *The Journal of Pathology* published by John Wiley & Sons Ltd on behalf of Pathological Society of Great Britain and Ireland.

p27^Kip1^ (p27, encoded by *CDKN1B*) is a CDK inhibitor of the Cip/Kip family which regulates CDK activity and has a vital role in controlling progression through the G1 phase of the cell cycle 1. Since its discovery, it has been assigned a multitude of additional biological functions including regulation of apoptosis, transcription and cell migration 1, 2. In accordance with its canonical role as a CDK inhibitor, it is well established that loss of p27 can contribute to the development of neoplastic disorders. Studies of *Cdkn1b*‐deficient mice indicated that p27 is haplo‐insufficient for tumour suppression. Animals lacking one or both alleles were found to be increasingly susceptible to carcinogen induced tumourigenesis, but the wild type allele was always retained in heterozygous animals 1, 2. This might hint towards a role of p27 in promoting tumour development. In addition, an increasing number of somatic and germline mutations have been identified and uncovered a role of *CDKN1B* as a tumour susceptibility gene in a number of human neoplasms 3. Consistent with these observations, low p27 is associated with poor prognosis in various cancer types. Alternatively, p27 can mislocalise to the cytoplasm in human tumours, where it is unavailable for inhibition of nuclear CDKs 1, 2.

Interestingly, a number of studies revealed that the role of p27 in oncogenesis is more complex than that of a simple tumour suppressor 1, 2, 3, 4 (Figure 1). For example, tyrosine phosphorylation impairs CDK inhibition by p27 and converts the inhibitor into an activating assembly factor of CDK4,6 that can promote cell proliferation 1 (Figure 1). Additional and CDK‐independent oncogenic functions of p27 have been proposed and were substantiated in a knock‐in mouse with a mutant p27 that is unable to bind cyclin/CDK complexes (p27^CK−^) and thereby loses its function as a tumour suppressor. Strikingly, this p27^CK−^ allele caused a dominant increase in spontaneous tumourigenesis compared to both wild type and *Cdkn1b* ^−/−^ knockout mice 5, 6. Whereas the complete loss of p27 caused spontaneous tumours only in the pituitary, expression of the p27^CK−^ allele caused tumours in multiple organs, including the lung 5, 6. In K‐Ras induced lung tumours, increased tumour numbers and aggressiveness were associated with increased Ras‐induced cytoplasmic localisation of p27^CK−^. In contrast, in the presence of c‐Myc, p27^CK−^ remained nuclear and did not contribute to c‐Myc induced transformation, suggesting that cytoplasmic localisation might be a prerequisite for the oncogenic activity of p27^CK−^ 6.

**Figure 1:**
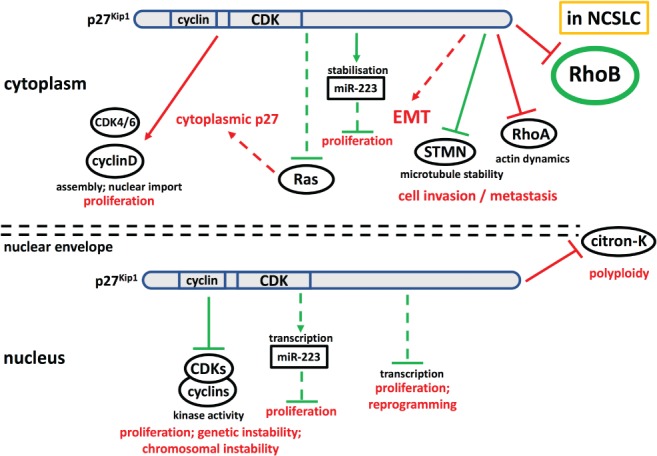
Schematic representation of the complex roles of p27^Kip1^ in tumourigenesis including RhoB as a novel target of cytoplasmic p27. Tumour suppressive activities are shown in green and oncogenic activities in red. Indirect actions are indicated by dashed lines. For more details see text and references 1, 2, 3, 4. citron‐K, citron kinase; EMT, epithelial mesenchymal transition; miR, micro‐RNA; STMN, stathmin.

p27 also plays a complex role in lung cancer, displaying both tumour suppressor and oncogenic activities. The growth of human non‐small cell lung carcinoma (NSCLC) cell lines carrying K‐Ras oncogenes requires interphase CDK activity. In particular, CDK4 was shown to be essential for the progression of K‐Ras driven NSCLC 7. Inhibition of CDK4 by nuclear p27 might be crucial in limiting lung tumourigenesis. Furthermore, cytoplasmic p27 was observed to be associated with a decrease in overall survival and unfavourable response to cisplatin‐based chemotherapy in human NSCLC 8. In a recent issue of *The Journal of Pathology*, Calvayrac *et al* uncovered a novel RhoB (Ras homolog gene family member B) dependent molecular mechanism that can explain an additional tumour‐promoting function of cytoplasmic p27 9.

Loss of RhoB expression is frequent in lung cancer, suggesting that it may have a tumour suppressor function in this tissue 10, 11. The three highly conserved Rho family GTPases RhoA, RhoB and RhoC share common roles in cytoskeletal reorganisation and cell motility 12. However, RhoB has a unique C‐terminus that is not only palmitoylated but can also be geranylgeranylated or farnesylated, which alters its function and localisation. Whereas farnesylated RhoB preferentially localises to the plasma membrane, geranylgeranylated RhoB can also localise to endosomes, multivesicular bodies and the nucleus. In most cancer types RhoA and RhoC are pro‐tumourigenic 12. The influence of RhoB activity on tumour development is less well studied and it appears that the small GTPase can play a two‐sided role. Its effect on tumour initiation and progression seems to depend on subcellular localisation and cellular context 12. Despite often being downregulated in lung cancer, RhoB seems to promote aggressive metastasis and resistance to therapy in lung adenocarcinoma 12.

Based on the observation that p27 can inhibit RhoA activation 1, 2, Calvayrac *et al* speculated that p27 might also be able to bind to a conserved region in RhoB and thereby prevent its activation. They demonstrate that this is indeed the case and proceed to show that p27 inhibits the interaction of RhoB with two RhoGEFs, p115 RhoGEF (ARHGEF1) and Lbc (AKAP13). As initially described for RhoA, this inhibition is also independent from the ability of p27 to bind CDKs and involves the C‐terminal eight amino acids of p27. These biochemical and cell biological observations were expanded with genetic evidence from mouse models, where Calvayrac and co‐workers ascertained that p27 and RhoB are linked in lung tumourigenesis. They speculated that the inhibition of RhoB by p27 might abrogate the selective pressure for RhoB loss in lung cancer. In support of this hypothesis, they observed that RhoB expression was preferentially lost in p27 knockout animals (64%), whereas RhoB remained more frequently expressed in mice expressing p27 or the p27 CK^−^ allele (40 or 31%, respectively). In addition, and consistent with their model, Calvayrac *et al* also observed that absence of RhoB enhanced the mean tumour size in p27^−/−^ animals, but had no effect on tumour number or size in p27 CK^−^ mice. This documents that at least some oncogenic activity of p27 is based on its inhibition of RhoB. It is somewhat unexpected that the combined deletion of RhoB and p27 leads only to significantly increased tumour volumes compared to p27 deletion alone, but did not significantly increase mean tumour numbers. One would have expected that, in the absence of p27, additional loss of RhoB would have a cumulative effect, if the tumour suppressor activities of the two proteins act on independent pathways. The lack of significantly increased tumour numbers indicates that inhibition of RhoB by p27 is surprisingly less important for tumour formation than for tumour growth.

The mouse model was based on urethane‐induced tumours that frequently involve activating mutations in K‐Ras (Q61 to R/L). Activated K‐Ras causes a fraction of p27 or p27^CK−^ to localise to the cytoplasm 13, potentially enhancing its interaction with RhoB. The susceptibility of mice to urethane‐induced carcinogenesis depends on genetic background where C57BL/6J mice are resistant to carcinogen induced lung tumourigenesis 14. As p27^−/−^ and RhoB^−/−^ transgenics were originally in different strains, Calvayrac *et al* generated a double heterozygous F1 generation in a mixed C57BL/6J/129S4 background. The F2 hybrids used in the experiments will carry some genomic heterogeneity that might also contribute to differences in the susceptibility to urethane‐induced tumourigenesis. However, a third independent approach further corroborated the link between cytoplasmic p27 and maintained RhoB expression, as it was observed in a patient cohort that cytoplasmic p27 and RhoB immunostaining associated with poor clinical outcomes, and that RhoB was preferentially lost in lung tumours that do not express p27. Together, these findings are exciting and open up the prospect that p27 and RhoB staining could be used as a biomarker in human lung cancer.

It remains to be determined if the lung cancer promoting activities of p27^CK−^ are solely due to RhoB inhibition. This could for example be addressed by a knock‐in model containing p27^CK−^ that cannot bind to RhoB. This may be challenging, since recent studies indicate that the interaction of p27 with the related RhoA protein may involve additional factors that bind to the p27 C‐terminus and a low affinity direct interaction of RhoA only with the p27 N‐terminus 15.

RhoB influences multiple cellular pathways including apoptosis, DNA damage response, cell cycle progression, migration and invasion. It will be interesting to determine the functions that are crucial for the tumour suppressive role of RhoB in NSCLC. While this study established a role of the RhoB/p27 axis in lung cancer, it will be important to elucidate if this mechanism also contributes to tumourigenesis in other organs and to uncover the crucial pathophysiological pathway affected by the RhoB/p27 axis.

## Author contributions statement

SRP, JV and LH were involved in writing and approving the final version of the manuscript.
